# Crystal structures of products from the BBr_3_-promoted de­methyl­ation of 3-(meth­oxymeth­yl)-6-phenyl-1,2,4,5-tetra­zine

**DOI:** 10.1107/S2056989026009527

**Published:** 2026-09-30

**Authors:** Jona Voss, Jan-Hendrik Lamm

**Affiliations:** a Bielefeld University, Department of Chemistry, Universitätsstr. 25, 33615 Bielefeld, Germany; Vienna University of Technology, Austria

**Keywords:** 1,2,4,5-tetra­zine, hydrogen bonds, crystal structure, synthesis

## Abstract

The BBr_3_-promoted de­methyl­ation of 3-(meth­oxy­meth­yl)-6-phenyl-1,2,4,5-tetra­zine, C_10_H_10_N_4_O, resulted in the alcohol (6-phenyl-1,2,4,5-tetrazin-3-yl)methanol, C_9_H_8_N_4_O, and the bromide 3-(bromo­meth­yl)-6-phenyl-1,2,4,5-tetra­zine, C_9_H_7_BrN_4_. All three compounds were analysed by nuclear magnetic resonance (NMR) spectroscopy in solution and by single-crystal X-ray diffraction using non-spherical scattering factors for structure refinement. The substrate as well as the corresponding bromide form π–π inter­actions between anti­parallel stacks of mol­ecules. In contrast, due to the inter­molecular hydrogen bonds present, which lead to the formation of a bent dimer, the alcohol forms only very weak parallel π–π stacks. Corresponding O—H⋯N hydrogen bonds are established between the hy­droxy group and an N atom of a neighbouring tetra­zine mol­ecule, an only rarely explored structural feature of 1,2,4,5-tetra­zines.

## Abstract 2

A (meth­oxy­meth­yl)-substituted 1,2,4,5-tetra­zine was treated with BBr_3_ to obtain both the (hy­droxy­meth­yl)- and (bromo­meth­yl)-substituted 1,2,4,5-tetra­zines; all three compounds were characterized by single-crystal X-ray diffraction measurements, which revealed the formation of inter­molecular π–π stacks for all tetra­zines, as well as hydrogen-bonded dimers of the tetra­zine alcohol.

## 1. Chemical context

1,2,4,5-Tetra­zines, first described by Pinner (1893 ▸), are electron-deficient heterocycles of particular relevance in fast and chemoselective cyclo­addition reactions (Zhao *et al.*, 2022 ▸). While structurally simple 1,2,4,5-tetra­zines (herein further abbreviated as tetra­zines) are well-known in the literature, for many modern applications, tetra­zines substituted at the 3- and 6-positions are needed (Yim *et al.*, 2026 ▸). In particular, tetra­zines modified with a hy­droxy­methyl group at the 3-position and derivatives thereof are frequently used as marker mol­ecules in biochemical applications (Baalmann *et al.*, 2019 ▸; Tu *et al.*, 2020 ▸; Wang *et al.*, 2022 ▸). Early reports on the synthesis and derivatization of such tetra­zines were provided by Lang *et al.* (1975 ▸) through the synthesis of a panel of 3,6-disubstituted tetra­zines. Out of this library, 3-(meth­oxy­meth­yl)-6-phenyl-1,2,4,5-tetra­zine (**1**) was synthesized (Tu *et al.*, 2019 ▸), and the BBr_3_-promoted de­methyl­ation re-investigated to obtain the deprotected alcohol (**2**) and the bromide (**3**) as a side product (Fig. 1 ▸). In the scope of the present investigations, tetra­zines (**1**)–(**3**) were characterised by NMR spectroscopy in solution and by single-crystal X-ray diffraction.
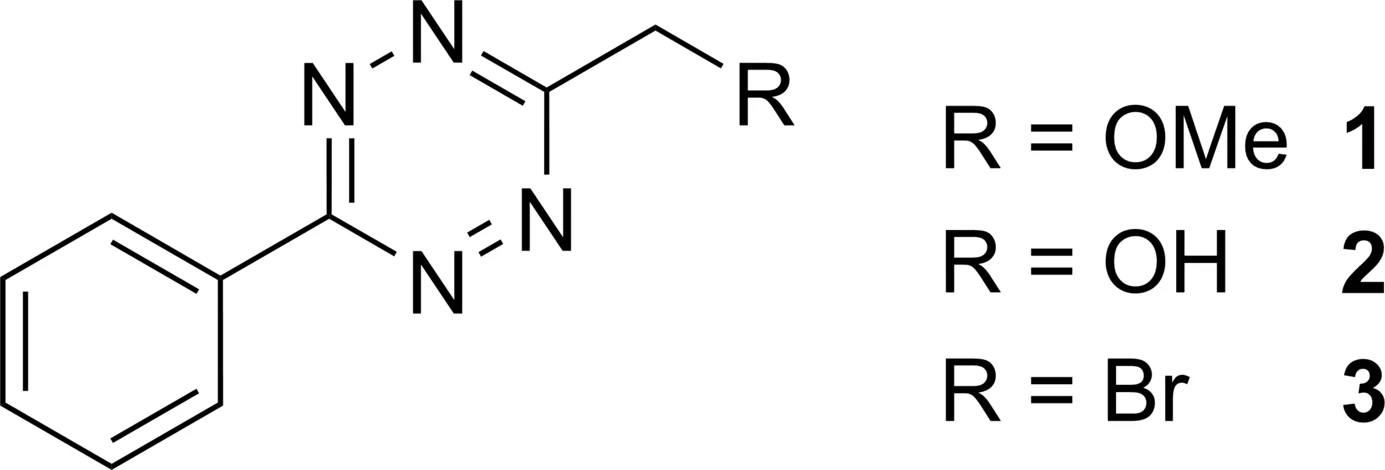


## 2. Structural commentary

While the structures of tetra­zine (**1**) and alcohol (**2**) were clearly confirmed by NMR spectroscopy in solution with ^13^C{^1^H} NMR shifts of the methyl­ene groups at 72.3 and 63.0 ppm, respectively, the strong upfield shift of the bromo­methyl group of bromide (**3**) to 27.8 ppm hints to the introduction of a strongly deshielding substituent. However, unambiguous structural proof was given by the results of single-crystal X-ray diffraction.

Both the substrate (**1**) and the bromide (**3**) crystallize in the triclinic space group *P*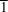 with one mol­ecule in the asymmetric unit each (Fig. 2 ▸). In contrast, alcohol (**2**) crystallizes in the monoclinic space group *P*2_1_/*n* with a hydrogen-bonded dimer in the asymmetric unit (Fig. 3 ▸). The two crystallographically independent mol­ecules (**2A**/**2B**) show close conformational similarity with a root-mean-square deviation of 0.169 Å (Fig. 3 ▸, right). The phenyl units of all tetra­zines (**1**)–(**3**) show similar bond lengths for all C—C bonds [1.391 (2)–1.401 (2) Å] and all C—N and N—N bonds of the tetra­zine units [1.308 (1)–1.353 (1) Å], indicative of their aromaticity with an extended co-planar arrangement of the biaryl moieties. The dihedral angles between the phenyl and tetra­zine rings are 3.63 (4)° (**1**), 2.29 (4)° (**2A**), 8.47 (5)° (**2B**), and 2.72 (1)° (**3**). All bond angles agree with values obtained from structurally similar disubstituted tetra­zines containing a phenyl and substituted methyl substituents (for details see Section 4). The meth­oxy­methyl group of tetra­zine (**1**) and the bromo­methyl group of bromide (**3**) accommodate the largest atoms (O and Br), resulting in the smallest torsion angles with 73.17 (8)° for the N1—C2—C1—O1 connection and −74.63 (17)° for the N4—C2—C1—Br1 connection, likely due to repulsion with the N-centered lone pairs of the tetra­zine units. Inter­estingly, the N1—C2—C1—O1 torsion angles of alcohol (**2**) are different from those of (**1**) and (**3**) with −162.92 (7)° for (**2A**) and 16.06 (9)° for (**2B**), an observation that may be explained by the inter­molecular hydrogen-bonding inter­actions between hy­droxy groups and tetra­zine-N atoms (see Section 3).

## 3. Supra­molecular features

All of the tetra­zines investigated here show π–π inter­actions, the strongest between anti­parallel stacked mol­ecules of tetra­zine (**1**) and bromide (**3**) with centroid-to-centroid distances of 3.724 (1) Å [slippage of 1.676 (1) Å] between the phen­yl/tetra­zine rings for (**1**) and of 3.716 (1) Å [slippage of 1.643 (3) Å] for (**3**)], respectively (Fig. 4 ▸).

Two hydrogen bonds between hy­droxy groups and tetra­zine N atoms of mol­ecules (**2A**) and (**2B**) form a bent dimer of alcohol (**2**), as shown in Fig. 5 ▸, bottom. The drastic reduction in the N1—C2—C1—O1 torsion angle compared with tetra­zine (**1**) may be explained by the reduced electron density at the neighbouring N atom, which is attributable to its role as a hydrogen-bond acceptor (Table 1 ▸). Similar hydrogen-bonding inter­actions were described previously for hy­droxy­methyl substituted tetra­zines (Voss *et al.*, 2025 ▸).

Inter­estingly, the inter­molecular hydrogen bonds of alcohol (**2**) cause the formation of weakly inter­acting parallel stacks of mol­ecules without contacts between phen­yl/tetra­zine ring centroids but with comparably elongated phen­yl/phenyl and tetra­zine/tetra­zine centroid-to-centroid distances of 4.557 (1) Å [slippage of 3.245 (1) Å] and 4.622 (1) Å [slippage of 3.311 (1) Å], respectively (Fig. 5 ▸, top). The observation of stronger inter­actions between anti­parallel stacks of tetra­zine (**1**) and the bromide (**3**) as compared to parallel stacks of alcohol (**2**) might be favoured by the increased dipole moment between phenyl and tetra­zine rings in anti­parallel stacks (Huber *et al.*, 2014 ▸; Voss *et al.*, 2025 ▸).

## 4. Database survey

A search of the Cambridge Structural Database (CSD version 6.01, November 2025; Groom *et al.*, 2016 ▸) using ConQuest (version 2026.1.0) revealed eight reports for 3-aryl-6-alkyl-1,2,4,5-tetra­zines, including CSD refcodes VEMZUM (Voss *et al.*, 2025 ▸), CICPOU (Hu & Xu, 2008 ▸) and REWDUT (Xu & Hu, 2007 ▸). Out of these, the lengths of all aromatic C—C, C—N and N—N bonds are identical within a 3*σ* margin of error. With dihedral angles between the phenyl and tetra­zine rings of tetra­zines (**1**)–(**3**) in the range 2.29 (4) to 8.47 (5)°, similar values between the two rings are also reported for these structurally similar tetra­zines.

## 5. Synthesis and crystallisation

### 5.1. Materials and methods

Solvents and starting materials were used without further purification, unless noted otherwise. Di­chloro­methane for reactions was dried by distillation over CaH_2_. The solvents used for extraction and chromatography were of technical grade and were distilled prior to use. Column chromatography was performed with silica gel 60 M (Acros, 0.04–0.063 mm). Reactions were monitored by thin-layer chromatography (TLC) carried out on silica gel plates (Merck, F254) using UV light (254 nm) for visualization. 3-(Meth­oxy­meth­yl)-6-phenyl-1,2,4,5-tetra­zine (**1**) was used as starting material and synthesised following a literature protocol (Tu *et al.*, 2019 ▸).

### 5.2. Analytical devices

Nuclear magnetic resonance spectra were recorded on an Avance 500 spectrometer (Bruker) at 298 K using the residual protonated solvent signal [*δ*(^1^H of CHCl_3_) = 7.26 ppm, *δ*(^13^C of CDCl_3_) = 77.36 ppm] (Fulmer *et al.*, 2010 ▸) as inter­nal standard. Electrospray ionisation (ESI) mass spectra (MS) were acquired with an Esquire 3000 ion trap mass spectrometer (Bruker Daltonik) equipped with a nano-ESI source.

### 5.3. De­methyl­ation of 3-(meth­oxy­meth­yl)-6-phenyl-1,2,4,5-tetra­zine (1)

This reaction was performed under an argon atmosphere. Tetra­zine (**1**) (593 mg, 2.93 mmol) was dissolved in dry CH_2_Cl_2_ (8 ml) and the resulting pink solution cooled to 258 K. A solution of BBr_3_ (0.70 ml, 7.38 mmol) in dry CH_2_Cl_2_ (15 ml) was added dropwise to this solution over the course of 10 min while maintaining the temperature. The resulting orange solution was stirred at 258 K for 105 min after which a small part of the solution (50 µl) was withdrawn, added to H_2_O (0.3 ml), and the organic layer analysed by TLC (CH_2_Cl_2_), which indicated full consumption of tetra­zine (**1**). After a total of 155 min, the reaction mixture was poured onto ice (50 g) and CH_2_Cl_2_ (100 ml) was added consecutively. After the ice had melted, the layers were separated and the aqueous layer extracted with CH_2_Cl_2_ (1×80 ml). The combined pink organic layers were washed with brine (40 ml), dried over MgSO_4_, filtered and evaporated to obtain a pink solid (481 mg). Purification by column chromatography (diameter = 4.5 cm, length = 23 cm) using CH_2_Cl_2_/MeOH (95:5, *v*/*v*) to firstly obtain bromide (**3**) (90.0 mg, 358 µmol, 12%) as a pink solid. Secondly, tetra­zine (**1**) (42.3 mg, 209 µmol, 7%) was re-isolated as a pink solid. Note that since analysis by TLC indicated complete consumption of tetra­zine (**1**) a small amount of it must have been formed during the work-up, likely by methyl­ation of alcohol (**2**) through *in situ* formed bromo­methane. Thirdly, a mixture of different compounds and alcohol (**2**) (333 mg, *R*_f_ (CH_2_Cl_2_/MeOH, 95:5, *v*/*v*) = 0.22–0.07) was obtained. Purification of the third fraction by column chromatography (diameter = 4.5 cm, length = 33 cm) using CH_2_Cl_2_/MeOH (97.5:2.5, *v*/*v*) obtained alcohol (**2**) (291 mg, 1.55 mmol, 53%) as a pink solid. In Lang’s pioneering work (Lang *et al.*, 1975 ▸), alcohol (**2**) was described as ‘relatively unstable’, a finding which we are unable to confirm – all the compounds isolated here remained stable in solid form at room temperature for over a year.

Analytical data of tetra­zine (**1**): ^1^H NMR (500 MHz, CDCl_3_) *δ* [ppm]: 8.63 (*m*, 2H, *o*-C^*ar­yl*^**H**), 7.67–7.57 (*m*, 3H, *m*-/*p*-C^*ar­yl*^**H**), 5.10 (*s*, 2H, C**H**_2_), 3.64 (*s*, 3H, C**H_3_**). ^13^C{^1^H}NMR (125 MHz{500 MHz}, CDCl_3_) *δ* [ppm]: 166.3 (**C**^*ar­yl*^CH_2_), 165.2 (**C**^*ar­yl*^Ph), 133.1 (*p*-**C**^*ar­yl*^), 131.7 (**C**^*ar­yl*^ attached to tetra­zine), 129.5 (*m*-**C**^*ar­yl*^), 128.4 (*o*-**C**^*ar­yl*^), 72.3 (**C**H_2_), 59.8 (**C**H_3_). MS (ESI, positive): *m*/*z =* 225.0 [C_10_H_10_N_4_O+Na]^+^, 427.1 [2·(C_10_H_10_N_4_O)+Na]^+^. *R*_f_ (CH_2_Cl_2_/MeOH, 95:5, *v*/*v*) = 0.25). Tetra­zine (**1**) was crystallized by slow evaporation from a CH_2_Cl_2_/MeOH solution at room temperature.

Analytical data of alcohol (**2**): ^1^H NMR (500 MHz, CDCl_3_) *δ* [ppm]: 8.62 (*m*, 2H, *o*-C^*ar­yl*^**H**), 7.66 (*m*, 1H, *p*-C^*ar­yl*^**H**), 7.61 (*m*, 2H, *m*-C^*ar­yl*^**H**), 5.34 (*d*, ^3^*J* = 6.2 Hz, 2H, C**H**_2_), 3.12 (*t*, ^3^*J* = 6.3 Hz, 1H, O**H**). ^13^C{^1^H}NMR (125 MHz{500 MHz}, CDCl_3_) *δ* [ppm]: 167.4 (**C**^*ar­yl*^CH_2_), 165.6 (**C**^*ar­yl*^Ph), 133.2 (*p*-**C**^*ar­yl*^), 131.6 (**C**^*ar­yl*^ attached to tetra­zine), 129.5 (*m*-**C**^*ar­yl*^), 128.4 (*o*-**C**^*ar­yl*^), 63.0 (**C**H_2_). MS (ESI, negative): *m*/*z =* 186.9 [C_9_H_8_N_4_O–H]^−^. *R*_f_ (CH_2_Cl_2_/MeOH, 97.5:2.5, *v*/*v*) = 0.20). Alcohol (**2**) was crystallized by slow evaporation from a CH_2_Cl_2_/cyclo­hexane solution at room temperature.

Analytical data of bromide (**3**): ^1^H NMR (500 MHz, CDCl_3_) *δ* [ppm]: 8.63 (*m*, 2H, *o*-C^*ar­yl*^**H**), 7.66 (*m*, 1H, *p*-C^*ar­yl*^**H**), 7.61 (*m*, 2H, *m*-C^*ar­yl*^**H**), 5.00 (*s*, 2H, C**H**_2_). ^13^C{^1^H}NMR (125 MHz{500 MHz}, CDCl_3_) *δ* [ppm]: 167.4 (**C**^*ar­yl*^CH_2_), 164.3 (**C**^*ar­yl*^Ph), 133.4 (*p*-**C**^*ar­yl*^), 131.4 (**C**^*ar­yl*^ attached to tetra­zine), 129.6 (*m*-**C**^*ar­yl*^), 128.6 (*o*-**C**^*ar­yl*^), 27.8 (**C**H_2_). MS (ESI): the compound was not ionisable. *R*_f_ (CH_2_Cl_2_/MeOH, 95:5, *v*/*v*) = 0.79. Bromide (**3**) was crystallized by slow evaporation from a CH_2_Cl_2_/MeOH solution at room temperature.

## 6. Refinement

Crystal data, data collection and structure refinement details are summarised in Table 2 ▸. All structures were refined using *NoSpherA2* (Kleemiss *et al.*, 2021 ▸), an implementation of non-spherical atom-form-factors in *OLEX2* (Dolomanov *et al.*, 2009 ▸). All non-hydrogen atoms were refined anisotropically; hydrogen atoms were refined isotropically. *ORCA 5.0* (Neese, 2022 ▸) was used for quantum mechanical calculations. The olex2_refinement_description section of the CIF gives further details.

## Supplementary Material

Crystal structure: contains datablock(s) global, 1, 2, 3. DOI: 10.1107/S2056989026009527/wm5811sup1.cif

Structure factors: contains datablock(s) 1. DOI: 10.1107/S2056989026009527/wm58111sup2.hkl

Structure factors: contains datablock(s) 2. DOI: 10.1107/S2056989026009527/wm58112sup3.hkl

Structure factors: contains datablock(s) 3. DOI: 10.1107/S2056989026009527/wm58113sup4.hkl

Supporting information file. DOI: 10.1107/S2056989026009527/wm58111sup5.cml

Supporting information file. DOI: 10.1107/S2056989026009527/wm58112sup6.cml

CCDC references: 2587346, 2587345, 2587344

Additional supporting information:  crystallographic information; 3D view; checkCIF report

## Figures and Tables

**Figure 1:**
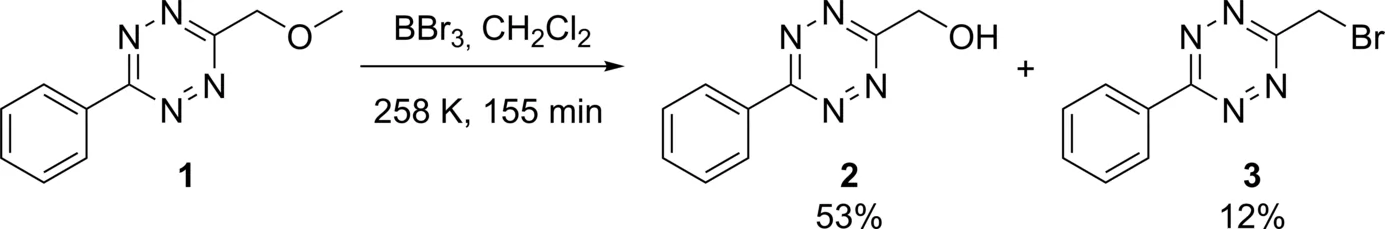
Treatment of tetra­zine (**1**) with BBr_3_ afforded both alcohol (**2**) and bromide (**3**).

**Figure 2:**
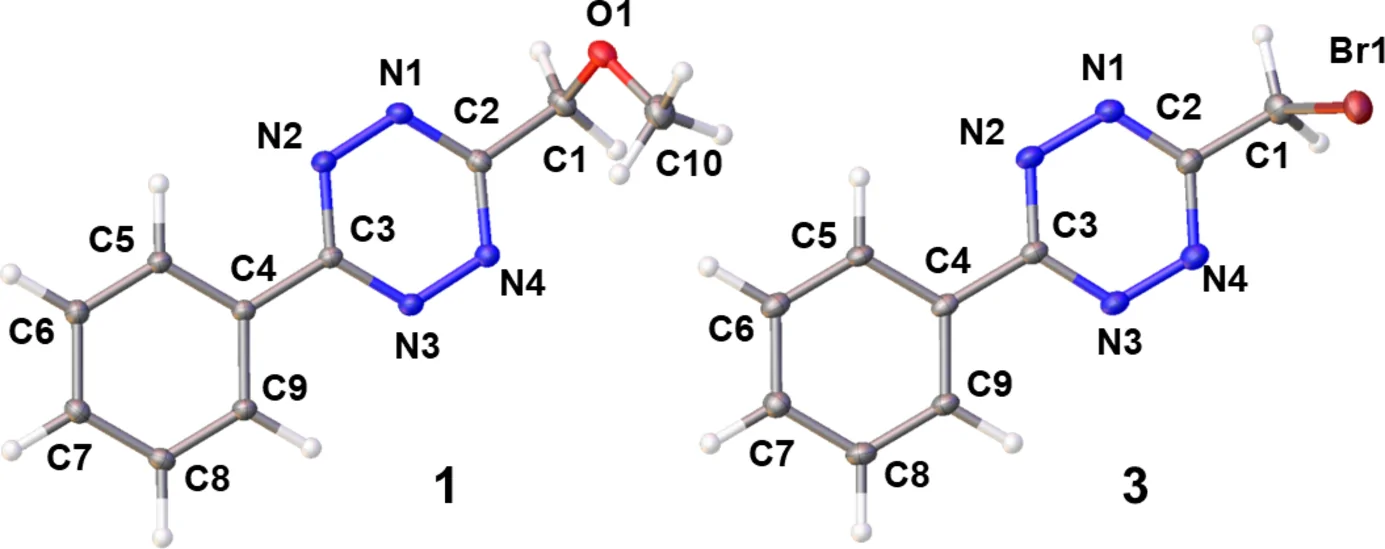
Mol­ecular structures of tetra­zine (**1**) and bromide (**3**) with displacement ellipsoids represented at the 50% probability level and H atoms as spheres of arbitrary radius.

**Figure 3:**
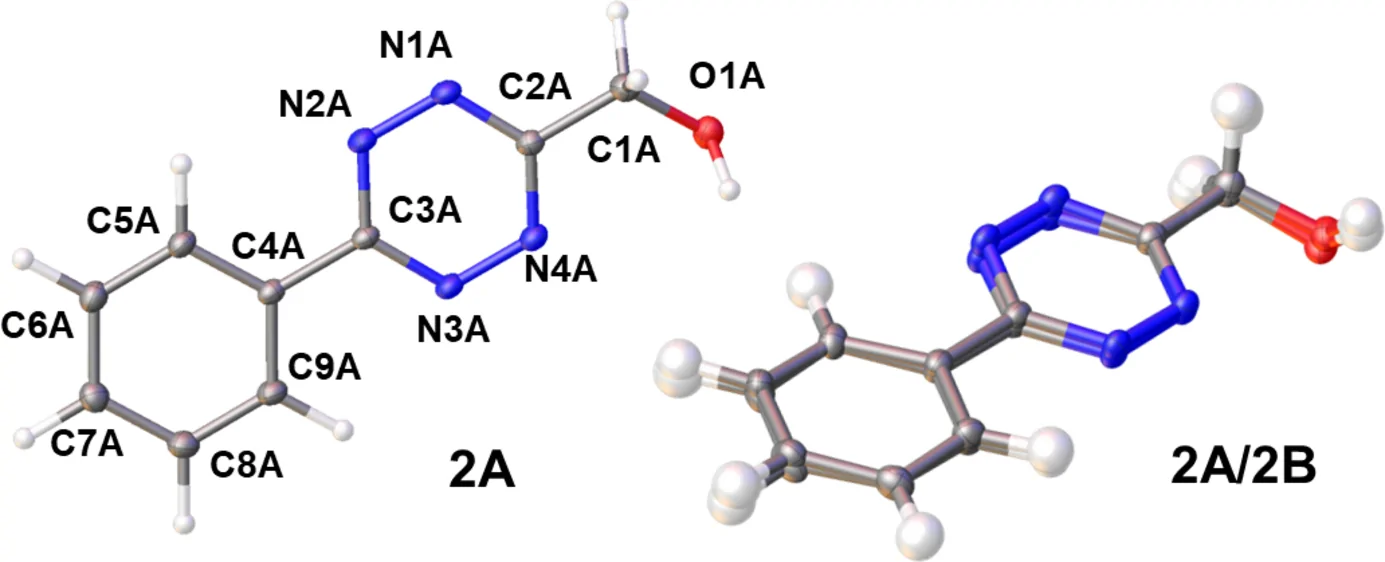
(Left) Mol­ecular structure of alcohol (**2**) with displacement ellipsoids represented at the 50% probability level and H atoms as spheres of arbitrary radius; for the sake of clarity only mol­ecule **A** is shown. (Right) Overlay-plot (generated using the ‘match’ option within *OLEX2*) of mol­ecules (**2A**/**2B**) showing their close conformational similarity.

**Figure 4:**
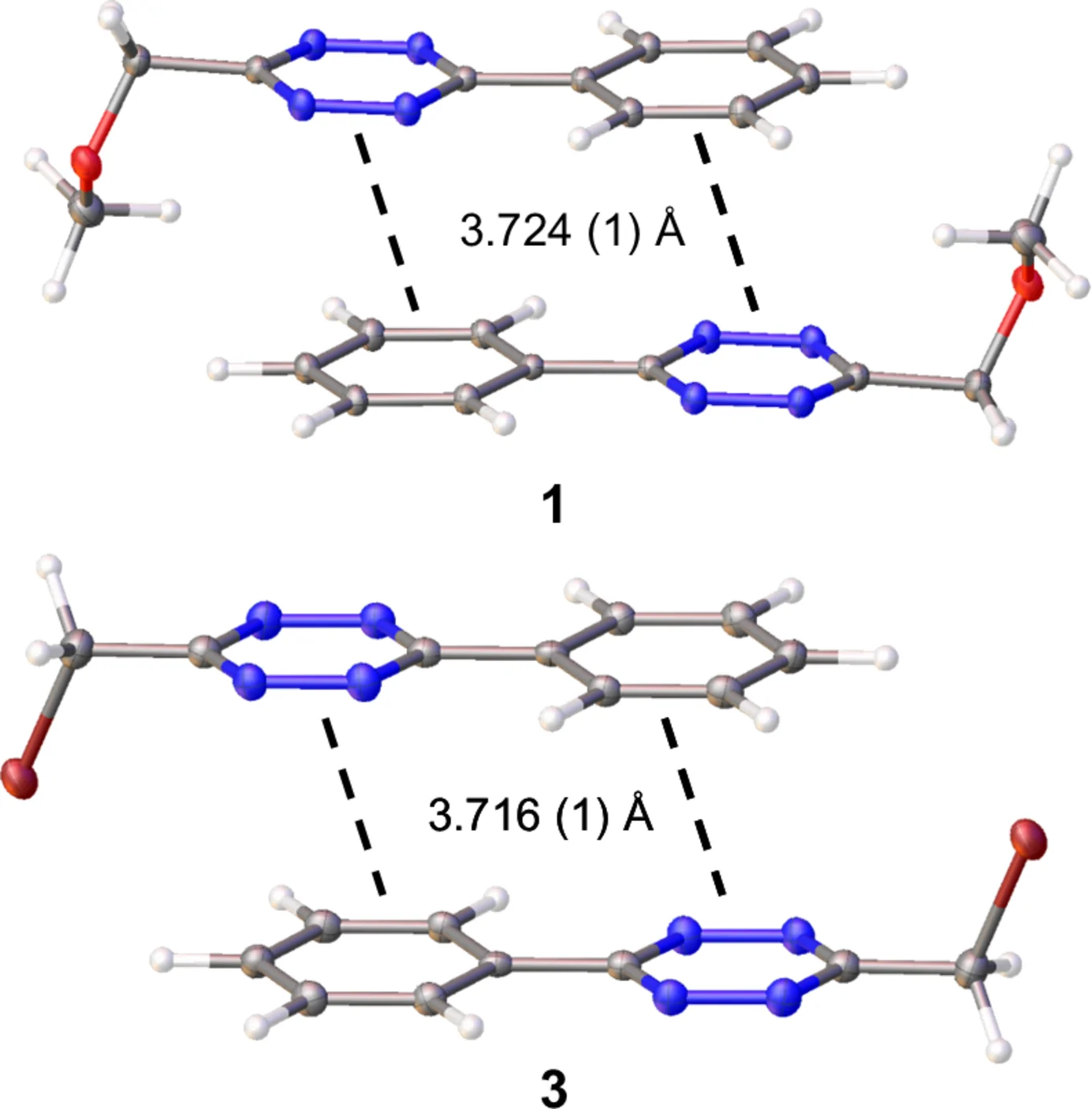
Supra­molecular π–π inter­actions of mol­ecules of tetra­zine (**1**) (top) and bromide (**3**) (bottom) with centroid-to-centroid distances indicated. Displacement ellipsoids are represented at the 50% probability level; symmetry code to obtain the second mol­ecule: (1 − *x*, 1 − *y*, 1 − *z*).

**Figure 5:**
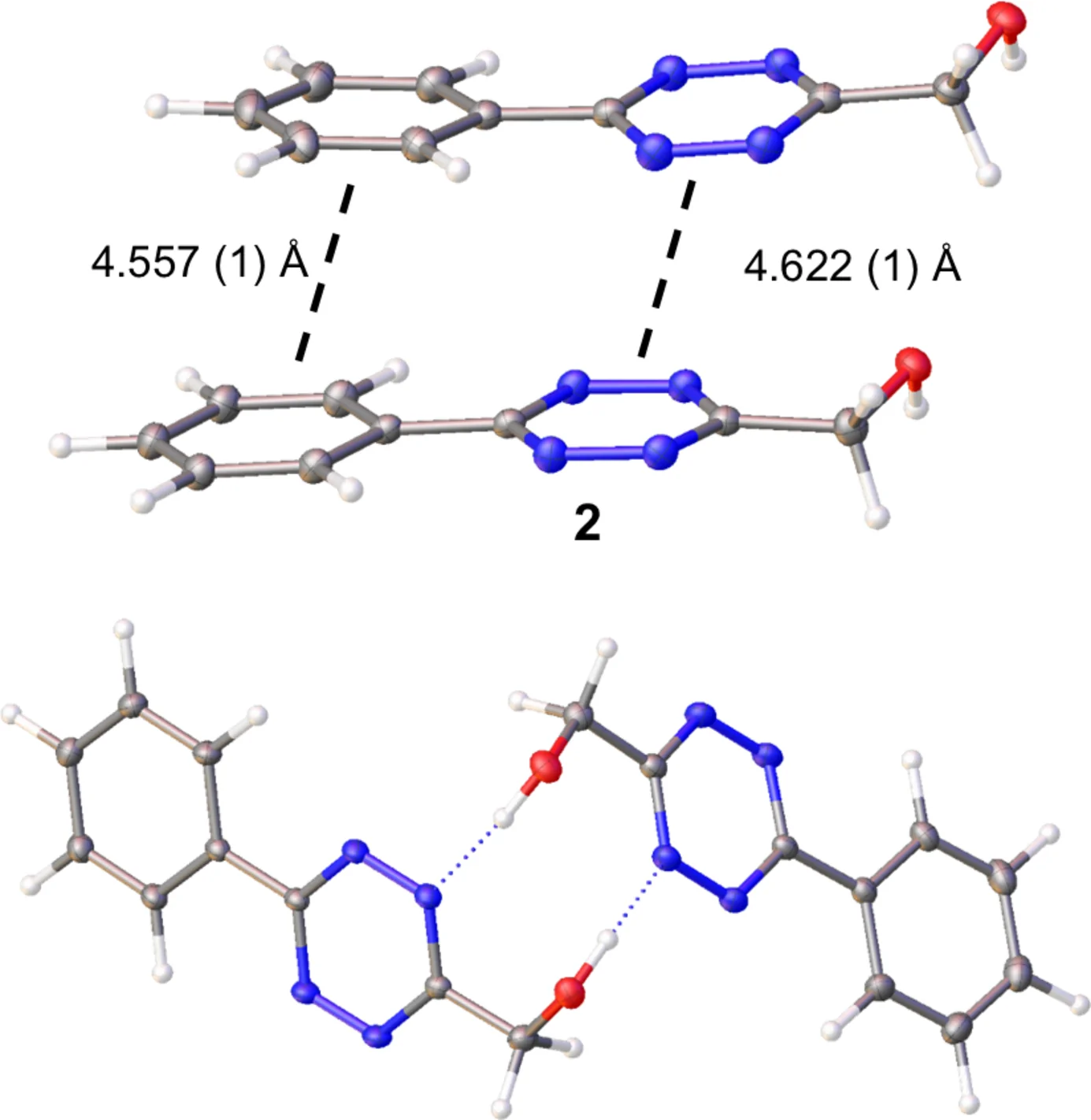
(Top) Supra­molecular π–π inter­actions of mol­ecules of alcohol (**2**) with the centroid-to-centroid distances indicated; (bottom) formation of a dimer through hydrogen bonds (dashed lines). Displacement ellipsoids are represented at the 50% probability level; symmetry code to obtain the second mol­ecule: (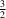 − *x*, 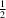 + *y*, 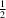 − *z*).

**Table 1:** Hydrogen-bond geometry (Å, °) for (**2**)

*D*—H⋯*A*	*D*—H	H⋯*A*	*D*⋯*A*	*D*—H⋯*A*
O1*A*—H1*A*⋯N1*B*	0.969 (12)	1.963 (13)	2.9019 (9)	162.7 (10)
O1*B*—H1*B*⋯N4*A*	0.972 (13)	1.871 (13)	2.8287 (9)	168.2 (11)

**Table 2:** Experimental details

	(**1**)	(**2**)	(**3**)
Crystal data			
Chemical formula	C_10_H_10_N_4_O	C_9_H_8_N_4_O	C_9_H_7_BrN_4_
*M*_r_	202.22	188.19	251.09
Crystal system, space group	Triclinic, *P* 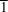	Monoclinic, *P*2_1_/*n*	Triclinic, *P* 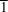
Temperature (K)	100	100	100
*a*, *b*, *c* (Å)	5.3147 (4), 7.4164 (8), 12.2277 (8)	11.8719 (3), 8.4682 (2), 17.2987 (5)	5.2376 (4), 7.5069 (6), 12.0638 (8)
α, β, γ (°)	86.710 (7), 79.913 (6), 78.920 (8)	90, 92.709 (2), 90	89.632 (6), 80.517 (6), 79.451 (7)
*V* (Å^3^)	465.53 (7)	1737.16 (8)	459.80 (6)
*Z*	2	8	2
Radiation type	Mo *K*α	Mo *K*α	Mo *K*α
μ (mm^−1^)	0.10	0.10	4.44
Crystal size (mm)	0.52 × 0.24 × 0.18	0.35 × 0.09 × 0.06	0.35 × 0.29 × 0.09

Data collection			
Diffractometer	SuperNova, Single source at offset/far, Eos	SuperNova, Single source at offset/far, Eos	SuperNova, Single source at offset/far, Eos
Absorption correction	Gaussian (*CrysAlis PRO*; Rigaku OD, 2025 ▸)	Gaussian (*CrysAlis PRO*; Rigaku OD, 2025 ▸)	Gaussian (*CrysAlis PRO*; Rigaku OD, 2025 ▸)
*T*_min_, *T*_max_	0.571, 1.000	0.744, 1.000	0.394, 1.000
No. of measured, independent and observed [*I* ≥ 2u(*I*)] reflections	8849, 3058, 2256	32315, 4666, 3774	7431, 2531, 2136
*R*_int_	0.035	0.045	0.048

Refinement			
*R*[*F*^2^ > 2σ(*F*^2^)], *wR*(*F*^2^), *S*	0.038, 0.061, 1.09	0.030, 0.066, 1.07	0.033, 0.061, 1.03
No. of reflections	3058	4666	2531
No. of parameters	166	317	148
H-atom treatment	H-atom parameters constrained	All H-atom parameters refined	H-atom parameters constrained
Δρ_max_, Δρ_min_ (e Å^−3^)	0.34, −0.33	0.21, −0.21	0.81, −0.70

Computer programs: *CrysAlis PRO* (Rigaku OD, 2025 ▸), *SHELXT* (Sheldrick, 2015 ▸), *OLEX2.refine* (Bourhis *et al.*, 2015 ▸), *OLEX2* (Dolomanov *et al.*, 2009 ▸) and *publCIF* (Westrip, 2010 ▸).

## supplementary crystallographic information

### 3-(Methoxymethyl)-6-phenyl-1,2,4,5-tetrazine (1) Crystal data

**Table:**

C_10_H_10_N_4_O	*Z* = 2
*M**_r_* = 202.22	*F*(000) = 212.144
Triclinic, *P*1	*D*_x_ = 1.443 Mg m^−^^3^
*a* = 5.3147 (4) Å	Mo *K*α radiation, λ = 0.71073 Å
*b* = 7.4164 (8) Å	Cell parameters from 3153 reflections
*c* = 12.2277 (8) Å	θ = 2.8–32.2°
α = 86.710 (7)°	µ = 0.10 mm^−^^1^
β = 79.913 (6)°	*T* = 100 K
γ = 78.920 (8)°	Block, clear light red
*V* = 465.53 (7) Å^3^	0.52 × 0.24 × 0.18 mm

### 3-(Methoxymethyl)-6-phenyl-1,2,4,5-tetrazine (1) Data collection

**Table:**

SuperNova, Single source at offset/far, Eos diffractometer	3058 independent reflections
Radiation source: micro-focus sealed X-ray tube, SuperNova (Mo) X-ray Source	2256 reflections with *I*≥ 2u(*I*)
Mirror monochromator	*R*_int_ = 0.035
Detector resolution: 8.0951 pixels mm^-1^	θ_max_ = 32.5°, θ_min_ = 1.7°
ω scans	*h* = −7→7
Absorption correction: gaussian (CrysAlisPro; Rigaku OD, 2025)	*k* = −10→10
*T*_min_ = 0.571, *T*_max_ = 1.000	*l* = −18→18
8849 measured reflections	

### 3-(Methoxymethyl)-6-phenyl-1,2,4,5-tetrazine (1) Refinement

**Table:**

Refinement on *F*^2^	10 constraints
Least-squares matrix: full	Primary atom site location: dual
*R*[*F*^2^ > 2σ(*F*^2^)] = 0.038	H-atom parameters constrained
*wR*(*F*^2^) = 0.061	*w* = 1/[σ^2^(*F*_o_^2^) + (0.0081*P*)^2^ + 0.0348*P*] where *P* = (*F*_o_^2^ + 2*F*_c_^2^)/3
*S* = 1.09	(Δ/σ)_max_ = −0.0002
3058 reflections	Δρ_max_ = 0.34 e Å^−^^3^
166 parameters	Δρ_min_ = −0.33 e Å^−^^3^
0 restraints	

### 3-(Methoxymethyl)-6-phenyl-1,2,4,5-tetrazine (1) Special details

**Table:**

Refinement. Refinement using NoSpherA2, an implementation of NOn-SPHERical Atom-form-factors in Olex2. The following options were used: SOFTWARE: ORCA 6.1 PARTITIONING: NoSpherA2 INT ACCURACY: Normal METHOD: r2SCAN BASIS SET: def2-TZVPP CHARGE: 0 MULTIPLICITY: 1 SOLVATION: Water DATE: 2026-09-01_14-23-46. Hydrogen atoms were refined isotropically.

### 3-(Methoxymethyl)-6-phenyl-1,2,4,5-tetrazine (1) Fractional atomic coordinates and isotropic or equivalent isotropic displacement parameters (Å^2^)

**Table:**

	*x*	*y*	*z*	*U*_iso_*/*U*_eq_	
O1	0.92532 (11)	0.72986 (10)	0.04592 (5)	0.01998 (16)	
N1	0.90879 (13)	0.72470 (11)	0.29459 (5)	0.01504 (16)	
N2	0.80917 (13)	0.68850 (11)	0.39730 (5)	0.01471 (16)	
N3	0.40354 (13)	0.86997 (11)	0.37086 (5)	0.01404 (16)	
N4	0.50426 (13)	0.90517 (11)	0.26685 (5)	0.01454 (16)	
C1	0.88144 (18)	0.88087 (14)	0.11730 (7)	0.01780 (19)	
H1A	1.0746 (17)	0.9105 (14)	0.1216 (7)	0.0214 (2)*	
H1B	0.7597 (17)	0.9979 (14)	0.0836 (7)	0.0214 (2)*	
C2	0.75552 (15)	0.83623 (13)	0.23290 (6)	0.01368 (17)	
C3	0.55900 (15)	0.76548 (12)	0.43439 (6)	0.01153 (17)	
C4	0.45400 (15)	0.73006 (12)	0.55162 (6)	0.01131 (17)	
C5	0.61259 (15)	0.61944 (13)	0.61873 (6)	0.01317 (18)	
H5	0.8094 (16)	0.5550 (13)	0.5846 (7)	0.0158 (2)*	
C6	0.51547 (16)	0.58844 (13)	0.73007 (7)	0.01524 (18)	
H6	0.6404 (16)	0.5014 (14)	0.7800 (7)	0.0183 (2)*	
C7	0.26109 (16)	0.66712 (13)	0.77537 (7)	0.01518 (18)	
H7	0.1901 (16)	0.6439 (13)	0.8630 (7)	0.0182 (2)*	
C8	0.10211 (16)	0.77628 (13)	0.70835 (7)	0.01495 (18)	
H8	−0.0982 (17)	0.8388 (14)	0.7428 (7)	0.0179 (2)*	
C9	0.19769 (15)	0.80708 (13)	0.59684 (7)	0.01359 (18)	
H9	0.0751 (16)	0.8899 (13)	0.5440 (7)	0.0163 (2)*	
C10	0.6980 (2)	0.70573 (16)	0.00716 (8)	0.0244 (2)	
H10A	0.7482 (19)	0.5807 (16)	−0.0412 (8)	0.0366 (3)*	
H10B	0.5439 (19)	0.7009 (15)	0.0746 (8)	0.0366 (3)*	
H10C	0.6313 (19)	0.8196 (16)	−0.0467 (8)	0.0366 (3)*	

### 3-(Methoxymethyl)-6-phenyl-1,2,4,5-tetrazine (1) Atomic displacement parameters (Å^2^)

**Table:**

	*U*^11^	*U*^22^	*U*^33^	*U*^12^	*U*^13^	*U*^23^
O1	0.0209 (3)	0.0204 (4)	0.0158 (3)	0.0008 (3)	0.0004 (2)	−0.0028 (3)
N1	0.0115 (3)	0.0168 (4)	0.0148 (3)	0.0001 (3)	0.0004 (3)	−0.0006 (3)
N2	0.0115 (3)	0.0165 (4)	0.0143 (3)	0.0009 (3)	−0.0012 (3)	0.0004 (3)
N3	0.0111 (3)	0.0158 (4)	0.0140 (3)	−0.0007 (3)	−0.0011 (3)	0.0007 (3)
N4	0.0132 (3)	0.0153 (4)	0.0137 (3)	−0.0005 (3)	−0.0014 (3)	0.0009 (3)
C1	0.0188 (4)	0.0172 (5)	0.0158 (4)	−0.0032 (4)	0.0011 (3)	0.0005 (4)
C2	0.0138 (4)	0.0131 (5)	0.0134 (4)	−0.0025 (3)	0.0000 (3)	−0.0005 (3)
C3	0.0103 (4)	0.0112 (4)	0.0130 (4)	−0.0019 (3)	−0.0018 (3)	−0.0007 (3)
C4	0.0102 (4)	0.0114 (4)	0.0123 (4)	−0.0019 (3)	−0.0015 (3)	−0.0007 (3)
C5	0.0114 (4)	0.0141 (5)	0.0134 (4)	−0.0010 (3)	−0.0018 (3)	−0.0006 (3)
C6	0.0142 (4)	0.0175 (5)	0.0135 (4)	−0.0018 (4)	−0.0025 (3)	0.0006 (3)
C7	0.0152 (4)	0.0167 (5)	0.0129 (4)	−0.0029 (4)	−0.0003 (3)	−0.0011 (3)
C8	0.0124 (4)	0.0167 (5)	0.0146 (4)	−0.0023 (4)	0.0008 (3)	−0.0014 (3)
C9	0.0108 (4)	0.0152 (5)	0.0140 (4)	−0.0011 (3)	−0.0017 (3)	−0.0001 (3)
C10	0.0280 (5)	0.0250 (6)	0.0199 (5)	−0.0030 (5)	−0.0043 (4)	−0.0032 (4)

### 3-(Methoxymethyl)-6-phenyl-1,2,4,5-tetrazine (1) Geometric parameters (Å, º)

**Table:**

O1—C1	1.4181 (11)	C4—C9	1.4003 (11)
O1—C10	1.4176 (11)	C5—H5	1.084 (8)
N1—N2	1.3087 (9)	C5—C6	1.3915 (11)
N1—C2	1.3422 (10)	C6—H6	1.078 (8)
N2—C3	1.3514 (10)	C6—C7	1.3952 (11)
N3—N4	1.3226 (9)	C7—H7	1.087 (9)
N3—C3	1.3403 (10)	C7—C8	1.3944 (11)
N4—C2	1.3359 (10)	C8—H8	1.096 (8)
C1—H1A	1.101 (9)	C8—C9	1.3912 (11)
C1—H1B	1.086 (9)	C9—H9	1.081 (8)
C1—C2	1.5019 (12)	C10—H10A	1.092 (11)
C3—C4	1.4724 (11)	C10—H10B	1.060 (10)
C4—C5	1.3986 (11)	C10—H10C	1.084 (11)
			
C10—O1—C1	113.11 (7)	C6—C5—C4	119.78 (7)
C2—N1—N2	117.77 (7)	C6—C5—H5	119.5 (4)
C3—N2—N1	118.01 (7)	H6—C6—C5	118.5 (5)
C3—N3—N4	117.89 (6)	C7—C6—C5	120.50 (8)
C2—N4—N3	117.60 (7)	C7—C6—H6	121.0 (5)
H1A—C1—O1	106.1 (5)	H7—C7—C6	119.4 (4)
H1B—C1—O1	109.8 (5)	C8—C7—C6	119.77 (8)
H1B—C1—H1A	111.3 (7)	C8—C7—H7	120.8 (4)
C2—C1—O1	112.18 (8)	H8—C8—C7	120.5 (5)
C2—C1—H1A	108.1 (5)	C9—C8—C7	119.99 (8)
C2—C1—H1B	109.4 (5)	C9—C8—H8	119.5 (5)
N4—C2—N1	124.67 (8)	C8—C9—C4	120.29 (8)
C1—C2—N1	115.93 (7)	H9—C9—C4	119.2 (5)
C1—C2—N4	119.39 (7)	H9—C9—C8	120.5 (5)
N3—C3—N2	123.89 (7)	H10A—C10—O1	107.4 (5)
C4—C3—N2	117.22 (7)	H10B—C10—O1	110.6 (5)
C4—C3—N3	118.89 (7)	H10B—C10—H10A	112.8 (9)
C5—C4—C3	119.64 (7)	H10C—C10—O1	111.3 (6)
C9—C4—C3	120.71 (7)	H10C—C10—H10A	108.2 (7)
C9—C4—C5	119.65 (7)	H10C—C10—H10B	106.6 (8)
H5—C5—C4	120.7 (4)		
			
O1—C1—C2—N1	73.17 (8)	N4—N3—C3—C4	177.76 (7)
O1—C1—C2—N4	−105.80 (8)	C2—N1—N2—C3	0.06 (9)
N1—N2—C3—N3	3.10 (9)	C2—N4—N3—C3	−1.00 (9)
N1—N2—C3—C4	−177.26 (7)	C2—C1—O1—C10	78.41 (9)
N1—C2—N4—N3	4.28 (11)	C3—C4—C5—C6	178.83 (8)
N2—N1—C2—N4	−3.79 (9)	C3—C4—C9—C8	−178.51 (8)
N2—N1—C2—C1	177.30 (8)	C4—C5—C6—C7	−0.10 (10)
N2—C3—N3—N4	−2.60 (10)	C4—C9—C8—C7	−0.51 (10)
N2—C3—C4—C5	−0.45 (9)	C5—C4—C9—C8	1.00 (10)
N2—C3—C4—C9	179.06 (8)	C5—C6—C7—C8	0.59 (11)
N3—N4—C2—C1	−176.84 (8)	C6—C5—C4—C9	−0.69 (10)
N3—C3—C4—C5	179.21 (8)	C6—C7—C8—C9	−0.29 (11)
N3—C3—C4—C9	−1.28 (10)		

### (6-Phenyl-1,2,4,5-tetrazin-3-yl)methanol (2) Crystal data

**Table:**

C_9_H_8_N_4_O	*F*(000) = 784.550
*M**_r_* = 188.19	*D*_x_ = 1.439 Mg m^−^^3^
Monoclinic, *P*2_1_/*n*	Mo *K*α radiation, λ = 0.71073 Å
*a* = 11.8719 (3) Å	Cell parameters from 9457 reflections
*b* = 8.4682 (2) Å	θ = 2.1–30.8°
*c* = 17.2987 (5) Å	µ = 0.10 mm^−^^1^
β = 92.709 (2)°	*T* = 100 K
*V* = 1737.16 (8) Å^3^	Needle, clear red
*Z* = 8	0.35 × 0.09 × 0.06 mm

### (6-Phenyl-1,2,4,5-tetrazin-3-yl)methanol (2) Data collection

**Table:**

SuperNova, Single source at offset/far, Eos diffractometer	4666 independent reflections
Radiation source: micro-focus sealed X-ray tube, SuperNova (Mo) X-ray Source	3774 reflections with *I*≥ 2u(*I*)
Mirror monochromator	*R*_int_ = 0.045
Detector resolution: 8.0951 pixels mm^-1^	θ_max_ = 29.1°, θ_min_ = 2.0°
ω scans	*h* = −16→17
Absorption correction: gaussian (CrysAlisPro; Rigaku OD, 2025)	*k* = −12→11
*T*_min_ = 0.744, *T*_max_ = 1.000	*l* = −24→24
32315 measured reflections	

### (6-Phenyl-1,2,4,5-tetrazin-3-yl)methanol (2) Refinement

**Table:**

Refinement on *F*^2^	0 constraints
Least-squares matrix: full	Primary atom site location: dual
*R*[*F*^2^ > 2σ(*F*^2^)] = 0.030	All H-atom parameters refined
*wR*(*F*^2^) = 0.066	*w* = 1/[σ^2^(*F*_o_^2^) + (0.0179*P*)^2^ + 0.2291*P*] where *P* = (*F*_o_^2^ + 2*F*_c_^2^)/3
*S* = 1.07	(Δ/σ)_max_ = −0.0000
4666 reflections	Δρ_max_ = 0.21 e Å^−^^3^
317 parameters	Δρ_min_ = −0.21 e Å^−^^3^
0 restraints	

### (6-Phenyl-1,2,4,5-tetrazin-3-yl)methanol (2) Special details

**Table:**

Refinement. Refinement using NoSpherA2, an implementation of NOn-SPHERical Atom-form-factors in Olex2. The following options were used: SOFTWARE: ORCA 6.1 PARTITIONING: NoSpherA2 INT ACCURACY: Normal METHOD: r2SCAN BASIS SET: def2-TZVPP CHARGE: 0 MULTIPLICITY: 1 SOLVATION: Water DATE: 2026-09-01_16-19-35. Hydrogen atoms were refined isotropically.

### (6-Phenyl-1,2,4,5-tetrazin-3-yl)methanol (2) Fractional atomic coordinates and isotropic or equivalent isotropic displacement parameters (Å^2^)

**Table:**

	*x*	*y*	*z*	*U*_iso_*/*U*_eq_	
O1A	0.59801 (5)	1.02413 (7)	0.28063 (4)	0.01965 (14)	
H1A	0.6607 (10)	0.9789 (14)	0.3110 (7)	0.029 (3)*	
O1B	0.86881 (5)	1.01966 (7)	0.25962 (4)	0.02058 (14)	
H1B	0.8062 (11)	0.9790 (15)	0.2273 (7)	0.035 (3)*	
N1A	0.52904 (6)	0.74303 (8)	0.12699 (4)	0.01679 (15)	
N1B	0.76371 (6)	0.82834 (8)	0.36380 (4)	0.01715 (15)	
N2A	0.57676 (5)	0.67508 (8)	0.06899 (4)	0.01688 (15)	
N2B	0.71656 (6)	0.75248 (8)	0.42088 (4)	0.01752 (15)	
N3A	0.75136 (6)	0.79518 (8)	0.10707 (4)	0.01599 (15)	
N3B	0.89047 (6)	0.62307 (9)	0.45041 (4)	0.01907 (16)	
N4A	0.70263 (6)	0.86196 (8)	0.16591 (4)	0.01601 (15)	
N4B	0.93624 (6)	0.69768 (9)	0.39344 (4)	0.01918 (16)	
C1A	0.53859 (7)	0.89889 (11)	0.24476 (5)	0.01957 (18)	
H1Aa	0.5285 (9)	0.8000 (13)	0.2849 (6)	0.039 (3)*	
H1Ab	0.4555 (9)	0.9394 (13)	0.2265 (6)	0.043 (3)*	
C1B	0.92960 (7)	0.88877 (11)	0.28883 (5)	0.01941 (18)	
H1Ba	0.9452 (9)	0.8015 (13)	0.2444 (6)	0.042 (3)*	
H1Bb	1.0106 (9)	0.9282 (12)	0.3117 (6)	0.034 (3)*	
C2A	0.59403 (6)	0.83232 (9)	0.17545 (5)	0.01452 (16)	
C2B	0.87263 (7)	0.80304 (10)	0.35268 (5)	0.01583 (17)	
C3A	0.68812 (6)	0.70005 (9)	0.06061 (5)	0.01377 (16)	
C3B	0.78189 (7)	0.65559 (10)	0.46492 (5)	0.01512 (17)	
C4A	0.74238 (6)	0.61986 (9)	−0.00351 (5)	0.01426 (16)	
C4B	0.73344 (7)	0.57847 (10)	0.53216 (5)	0.01579 (17)	
C5A	0.67944 (7)	0.52052 (10)	−0.05393 (5)	0.01702 (17)	
H5A	0.5903 (8)	0.5023 (12)	−0.0456 (6)	0.031 (3)*	
C5B	0.61878 (7)	0.59683 (11)	0.54546 (5)	0.02024 (18)	
H5B	0.5665 (9)	0.6653 (14)	0.5054 (6)	0.040 (3)*	
C6A	0.73229 (7)	0.44423 (10)	−0.11365 (5)	0.01900 (18)	
H6A	0.6838 (9)	0.3643 (13)	−0.1510 (6)	0.036 (3)*	
C6B	0.57384 (8)	0.52971 (11)	0.61061 (5)	0.0245 (2)	
H6B	0.4841 (9)	0.5440 (13)	0.6203 (6)	0.039 (3)*	
C7A	0.84716 (7)	0.46702 (11)	−0.12364 (5)	0.01942 (18)	
H7A	0.8874 (9)	0.4069 (13)	−0.1685 (7)	0.043 (3)*	
C7B	0.64302 (8)	0.44385 (12)	0.66256 (5)	0.0257 (2)	
H7B	0.6084 (9)	0.3926 (13)	0.7137 (7)	0.041 (3)*	
C8A	0.90950 (7)	0.56615 (11)	−0.07371 (5)	0.01958 (18)	
H8A	0.9988 (9)	0.5834 (13)	−0.0805 (6)	0.038 (3)*	
C8B	0.75664 (8)	0.42286 (11)	0.64873 (5)	0.0247 (2)	
H8B	0.8100 (9)	0.3533 (14)	0.6870 (7)	0.048 (3)*	
C9A	0.85757 (7)	0.64189 (10)	−0.01341 (5)	0.01796 (18)	
H9A	0.9041 (8)	0.7182 (12)	0.0273 (6)	0.028 (3)*	
C9B	0.80233 (7)	0.48971 (10)	0.58373 (5)	0.01967 (18)	
H9B	0.8907 (9)	0.4737 (13)	0.5731 (6)	0.039 (3)*	

### (6-Phenyl-1,2,4,5-tetrazin-3-yl)methanol (2) Atomic displacement parameters (Å^2^)

**Table:**

	*U*^11^	*U*^22^	*U*^33^	*U*^12^	*U*^13^	*U*^23^
O1A	0.0218 (3)	0.0163 (3)	0.0205 (3)	0.0027 (2)	−0.0014 (3)	−0.0006 (2)
O1B	0.0229 (3)	0.0159 (3)	0.0225 (3)	−0.0039 (2)	−0.0036 (3)	−0.0006 (2)
N1A	0.0115 (3)	0.0197 (4)	0.0190 (4)	−0.0028 (3)	−0.0002 (3)	−0.0001 (3)
N1B	0.0151 (3)	0.0180 (4)	0.0182 (4)	0.0037 (3)	−0.0015 (3)	0.0009 (3)
N2A	0.0120 (3)	0.0199 (4)	0.0187 (4)	−0.0036 (3)	−0.0004 (3)	−0.0019 (3)
N2B	0.0140 (3)	0.0197 (4)	0.0188 (4)	0.0044 (3)	−0.0006 (3)	0.0011 (3)
N3A	0.0125 (3)	0.0189 (4)	0.0164 (4)	−0.0045 (3)	−0.0005 (3)	−0.0008 (3)
N3B	0.0149 (3)	0.0211 (4)	0.0210 (4)	0.0054 (3)	−0.0016 (3)	0.0015 (3)
N4A	0.0145 (3)	0.0174 (4)	0.0160 (3)	−0.0038 (3)	−0.0006 (3)	−0.0005 (3)
N4B	0.0145 (3)	0.0215 (4)	0.0215 (4)	0.0035 (3)	−0.0000 (3)	0.0010 (3)
C1A	0.0161 (4)	0.0211 (5)	0.0217 (5)	0.0003 (3)	0.0019 (3)	−0.0022 (4)
C1B	0.0196 (4)	0.0174 (4)	0.0212 (5)	−0.0018 (3)	0.0007 (3)	−0.0013 (3)
C2A	0.0132 (4)	0.0141 (4)	0.0162 (4)	−0.0005 (3)	−0.0003 (3)	0.0010 (3)
C2B	0.0146 (4)	0.0154 (4)	0.0173 (4)	0.0007 (3)	−0.0012 (3)	−0.0018 (3)
C3A	0.0117 (4)	0.0151 (4)	0.0144 (4)	−0.0025 (3)	−0.0008 (3)	0.0015 (3)
C3B	0.0141 (4)	0.0151 (4)	0.0160 (4)	0.0020 (3)	−0.0014 (3)	−0.0018 (3)
C4A	0.0131 (4)	0.0156 (4)	0.0139 (4)	−0.0026 (3)	−0.0009 (3)	0.0015 (3)
C4B	0.0166 (4)	0.0149 (4)	0.0157 (4)	0.0011 (3)	−0.0013 (3)	−0.0017 (3)
C5A	0.0152 (4)	0.0197 (4)	0.0160 (4)	−0.0035 (3)	−0.0009 (3)	−0.0005 (3)
C5B	0.0176 (4)	0.0230 (5)	0.0202 (5)	0.0023 (3)	0.0013 (3)	−0.0010 (4)
C6A	0.0198 (4)	0.0207 (4)	0.0164 (4)	−0.0035 (3)	−0.0003 (3)	−0.0017 (3)
C6B	0.0234 (5)	0.0287 (5)	0.0217 (5)	−0.0027 (4)	0.0048 (4)	−0.0015 (4)
C7A	0.0201 (4)	0.0219 (4)	0.0165 (4)	0.0002 (3)	0.0029 (3)	−0.0004 (3)
C7B	0.0320 (5)	0.0267 (5)	0.0183 (5)	−0.0086 (4)	0.0015 (4)	0.0006 (4)
C8A	0.0147 (4)	0.0245 (5)	0.0196 (4)	−0.0009 (3)	0.0022 (3)	−0.0002 (3)
C8B	0.0289 (5)	0.0243 (5)	0.0203 (5)	−0.0049 (4)	−0.0057 (4)	0.0039 (4)
C9A	0.0134 (4)	0.0221 (4)	0.0183 (4)	−0.0030 (3)	0.0002 (3)	−0.0010 (3)
C9B	0.0201 (4)	0.0194 (4)	0.0190 (4)	−0.0005 (3)	−0.0043 (3)	0.0012 (3)

### (6-Phenyl-1,2,4,5-tetrazin-3-yl)methanol (2) Geometric parameters (Å, º)

**Table:**

O1A—H1A	0.969 (12)	C3B—C4B	1.4742 (12)
O1A—C1A	1.4022 (10)	C4A—C5A	1.4018 (11)
O1B—H1B	0.972 (13)	C4A—C9A	1.3988 (11)
O1B—C1B	1.4036 (10)	C4B—C5B	1.3999 (11)
N1A—N2A	1.3086 (10)	C4B—C9B	1.4003 (11)
N1A—C2A	1.3448 (10)	C5A—H5A	1.085 (10)
N1B—N2B	1.3242 (10)	C5A—C6A	1.3926 (12)
N1B—C2B	1.3336 (10)	C5B—H5B	1.078 (11)
N2A—C3A	1.3534 (10)	C5B—C6B	1.3909 (13)
N2B—C3B	1.3413 (10)	C6A—H6A	1.082 (10)
N3A—N4A	1.3214 (10)	C6A—C7A	1.3961 (12)
N3A—C3A	1.3421 (10)	C6B—H6B	1.093 (11)
N3B—N4B	1.3101 (10)	C6B—C7B	1.3932 (13)
N3B—C3B	1.3533 (10)	C7A—H7A	1.061 (11)
N4A—C2A	1.3314 (10)	C7A—C8A	1.3922 (12)
N4B—C2B	1.3466 (10)	C7B—H7B	1.084 (11)
C1A—H1Aa	1.098 (11)	C7B—C8B	1.3925 (13)
C1A—H1Ab	1.078 (11)	C8A—H8A	1.082 (10)
C1A—C2A	1.5042 (12)	C8A—C9A	1.3929 (12)
C1B—H1Ba	1.089 (11)	C8B—H8B	1.071 (11)
C1B—H1Bb	1.075 (10)	C8B—C9B	1.3918 (13)
C1B—C2B	1.5083 (12)	C9A—H9A	1.087 (10)
C3A—C4A	1.4745 (11)	C9B—H9B	1.082 (11)
			
C1A—O1A—H1A	107.3 (7)	C9A—C4A—C3A	119.68 (7)
C1B—O1B—H1B	107.0 (7)	C9A—C4A—C5A	119.95 (8)
C2A—N1A—N2A	117.96 (7)	C5B—C4B—C3B	119.95 (7)
C2B—N1B—N2B	118.46 (7)	C9B—C4B—C3B	120.17 (7)
C3A—N2A—N1A	118.29 (7)	C9B—C4B—C5B	119.87 (8)
C3B—N2B—N1B	117.83 (7)	H5A—C5A—C4A	119.8 (5)
C3A—N3A—N4A	117.73 (7)	C6A—C5A—C4A	119.63 (8)
C3B—N3B—N4B	118.29 (7)	C6A—C5A—H5A	120.6 (5)
C2A—N4A—N3A	118.46 (7)	H5B—C5B—C4B	119.3 (6)
C2B—N4B—N3B	118.11 (7)	C6B—C5B—C4B	120.07 (8)
H1Aa—C1A—O1A	111.4 (6)	C6B—C5B—H5B	120.6 (6)
H1Ab—C1A—O1A	108.9 (6)	H6A—C6A—C5A	119.1 (6)
H1Ab—C1A—H1Aa	107.6 (8)	C7A—C6A—C5A	120.31 (8)
C2A—C1A—O1A	113.97 (7)	C7A—C6A—H6A	120.5 (6)
C2A—C1A—H1Aa	106.4 (6)	H6B—C6B—C5B	119.6 (6)
C2A—C1A—H1Ab	108.3 (6)	C7B—C6B—C5B	119.93 (9)
H1Ba—C1B—O1B	112.6 (6)	C7B—C6B—H6B	120.5 (6)
H1Bb—C1B—O1B	108.8 (6)	H7A—C7A—C6A	120.0 (6)
H1Bb—C1B—H1Ba	106.9 (8)	C8A—C7A—C6A	120.02 (8)
C2B—C1B—O1B	113.76 (7)	C8A—C7A—H7A	119.9 (6)
C2B—C1B—H1Ba	106.7 (6)	H7B—C7B—C6B	120.1 (6)
C2B—C1B—H1Bb	107.7 (6)	C8B—C7B—C6B	120.14 (9)
N4A—C2A—N1A	123.99 (8)	C8B—C7B—H7B	119.8 (6)
C1A—C2A—N1A	116.60 (7)	H8A—C8A—C7A	120.6 (6)
C1A—C2A—N4A	119.40 (7)	C9A—C8A—C7A	120.08 (8)
N4B—C2B—N1B	123.68 (8)	C9A—C8A—H8A	119.3 (6)
C1B—C2B—N1B	119.98 (7)	H8B—C8B—C7B	120.9 (6)
C1B—C2B—N4B	116.27 (7)	C9B—C8B—C7B	120.31 (9)
N3A—C3A—N2A	123.45 (8)	C9B—C8B—H8B	118.8 (6)
C4A—C3A—N2A	118.18 (7)	C8A—C9A—C4A	120.01 (8)
C4A—C3A—N3A	118.36 (7)	H9A—C9A—C4A	118.0 (5)
N3B—C3B—N2B	123.33 (8)	H9A—C9A—C8A	122.0 (5)
C4B—C3B—N2B	118.84 (7)	C8B—C9B—C4B	119.66 (8)
C4B—C3B—N3B	117.83 (7)	H9B—C9B—C4B	120.0 (6)
C5A—C4A—C3A	120.37 (7)	H9B—C9B—C8B	120.3 (6)
			
O1A—C1A—C2A—N1A	−162.92 (7)	N3B—C3B—C4B—C5B	−174.15 (7)
O1A—C1A—C2A—N4A	18.03 (9)	N3B—C3B—C4B—C9B	7.26 (9)
O1B—C1B—C2B—N1B	16.06 (9)	N4A—N3A—C3A—C4A	−177.69 (7)
O1B—C1B—C2B—N4B	−166.93 (7)	N4B—N3B—C3B—C4B	−176.25 (7)
N1A—N2A—C3A—N3A	−2.39 (9)	C2A—N1A—N2A—C3A	−0.78 (9)
N1A—N2A—C3A—C4A	178.34 (7)	C2A—N4A—N3A—C3A	−0.49 (8)
N1A—C2A—N4A—N3A	−2.73 (9)	C2B—N1B—N2B—C3B	0.77 (9)
N1B—N2B—C3B—N3B	−5.01 (9)	C2B—N4B—N3B—C3B	0.23 (9)
N1B—N2B—C3B—C4B	175.76 (7)	C3A—C4A—C5A—C6A	−179.25 (8)
N1B—C2B—N4B—N3B	−4.47 (10)	C3A—C4A—C9A—C8A	179.81 (8)
N2A—N1A—C2A—N4A	3.38 (9)	C3B—C4B—C5B—C6B	−177.30 (8)
N2A—N1A—C2A—C1A	−175.62 (7)	C3B—C4B—C9B—C8B	177.35 (8)
N2A—C3A—N3A—N4A	3.04 (9)	C4A—C5A—C6A—C7A	−0.35 (10)
N2A—C3A—C4A—C5A	−0.12 (9)	C4A—C9A—C8A—C7A	−0.75 (10)
N2A—C3A—C4A—C9A	−179.41 (7)	C4B—C5B—C6B—C7B	−0.14 (10)
N2B—N1B—C2B—N4B	3.95 (9)	C4B—C9B—C8B—C7B	0.03 (10)
N2B—N1B—C2B—C1B	−179.28 (7)	C5A—C4A—C9A—C8A	0.51 (9)
N2B—C3B—N3B—N4B	4.50 (9)	C5A—C6A—C7A—C8A	0.12 (10)
N2B—C3B—C4B—C5B	5.13 (9)	C5B—C4B—C9B—C8B	−1.23 (9)
N2B—C3B—C4B—C9B	−173.46 (8)	C5B—C6B—C7B—C8B	−1.07 (11)
N3A—N4A—C2A—C1A	176.25 (7)	C6A—C5A—C4A—C9A	0.04 (10)
N3A—C3A—C4A—C5A	−179.42 (7)	C6A—C7A—C8A—C9A	0.43 (10)
N3A—C3A—C4A—C9A	1.29 (9)	C6B—C5B—C4B—C9B	1.29 (10)
N3B—N4B—C2B—C1B	178.65 (7)	C6B—C7B—C8B—C9B	1.13 (11)

### (6-Phenyl-1,2,4,5-tetrazin-3-yl)methanol (2) Hydrogen-bond geometry (Å, º)

**Table:**

*D*—H···*A*	*D*—H	H···*A*	*D*···*A*	*D*—H···*A*
O1*A*—H1*A*···N1*B*	0.969 (12)	1.963 (13)	2.9019 (9)	162.7 (10)
O1*B*—H1*B*···N4*A*	0.972 (13)	1.871 (13)	2.8287 (9)	168.2 (11)

### 3-(Bromomethyl)-6-phenyl-1,2,4,5-tetrazine (3) Crystal data

**Table:**

C_9_H_7_BrN_4_	*Z* = 2
*M**_r_* = 251.09	*F*(000) = 247.736
Triclinic, *P*1	*D*_x_ = 1.814 Mg m^−^^3^
*a* = 5.2376 (4) Å	Mo *K*α radiation, λ = 0.71073 Å
*b* = 7.5069 (6) Å	Cell parameters from 3207 reflections
*c* = 12.0638 (8) Å	θ = 3.2–30.6°
α = 89.632 (6)°	µ = 4.44 mm^−^^1^
β = 80.517 (6)°	*T* = 100 K
γ = 79.451 (7)°	Block, clear red
*V* = 459.80 (6) Å^3^	0.35 × 0.29 × 0.09 mm

### 3-(Bromomethyl)-6-phenyl-1,2,4,5-tetrazine (3) Data collection

**Table:**

SuperNova, Single source at offset/far, Eos diffractometer	2531 independent reflections
Radiation source: micro-focus sealed X-ray tube, SuperNova (Mo) X-ray Source	2136 reflections with *I*≥ 2u(*I*)
Mirror monochromator	*R*_int_ = 0.048
Detector resolution: 8.0951 pixels mm^-1^	θ_max_ = 29.6°, θ_min_ = 1.7°
ω scans	*h* = −7→7
Absorption correction: gaussian (CrysAlisPro; Rigaku OD, 2025)	*k* = −10→10
*T*_min_ = 0.394, *T*_max_ = 1.000	*l* = −17→16
7431 measured reflections	

### 3-(Bromomethyl)-6-phenyl-1,2,4,5-tetrazine (3) Refinement

**Table:**

Refinement on *F*^2^	7 constraints
Least-squares matrix: full	Primary atom site location: dual
*R*[*F*^2^ > 2σ(*F*^2^)] = 0.033	H-atom parameters constrained
*wR*(*F*^2^) = 0.061	*w* = 1/[σ^2^(*F*_o_^2^) + (0.0159*P*)^2^] where *P* = (*F*_o_^2^ + 2*F*_c_^2^)/3
*S* = 1.03	(Δ/σ)_max_ = −0.0002
2531 reflections	Δρ_max_ = 0.81 e Å^−^^3^
148 parameters	Δρ_min_ = −0.70 e Å^−^^3^
0 restraints	

### 3-(Bromomethyl)-6-phenyl-1,2,4,5-tetrazine (3) Special details

**Table:**

Refinement. Refinement using NoSpherA2, an implementation of NOn-SPHERical Atom-form-factors in Olex2. The following options were used: SOFTWARE: ORCA 6.1 PARTITIONING: NoSpherA2 INT ACCURACY: Normal METHOD: r2SCAN BASIS SET: def2-TZVPP CHARGE: 0 MULTIPLICITY: 1 SOLVATION: Water DATE: 2026-09-01_14-39-25. Hydrogen atoms were refined isotropically.

### 3-(Bromomethyl)-6-phenyl-1,2,4,5-tetrazine (3) Fractional atomic coordinates and isotropic or equivalent isotropic displacement parameters (Å^2^)

**Table:**

	*x*	*y*	*z*	*U*_iso_*/*U*_eq_	
Br1	0.65100 (5)	0.72768 (3)	0.010906 (19)	0.02391 (8)	
N1	0.8794 (3)	0.7258 (3)	0.28825 (15)	0.0176 (4)	
N2	0.7978 (3)	0.6944 (3)	0.39466 (15)	0.0178 (4)	
N3	0.3775 (3)	0.8719 (2)	0.38276 (15)	0.0172 (4)	
N4	0.4589 (3)	0.9025 (3)	0.27664 (16)	0.0191 (4)	
C1	0.8027 (5)	0.8656 (3)	0.11273 (19)	0.0212 (5)	
H1a	1.023 (5)	0.823 (3)	0.0968 (19)	0.0254 (6)*	
H1b	0.726 (5)	1.015 (3)	0.097 (2)	0.0254 (6)*	
C2	0.7078 (4)	0.8275 (3)	0.23224 (18)	0.0159 (4)	
C3	0.5477 (4)	0.7671 (3)	0.43979 (18)	0.0144 (4)	
C4	0.4543 (4)	0.7315 (3)	0.55885 (17)	0.0134 (4)	
C5	0.6212 (4)	0.6175 (3)	0.62007 (18)	0.0148 (4)	
H5	0.817 (4)	0.550 (3)	0.5826 (18)	0.0178 (5)*	
C6	0.5325 (4)	0.5841 (3)	0.73262 (19)	0.0174 (5)	
H6	0.664 (4)	0.495 (3)	0.7779 (19)	0.0209 (5)*	
C7	0.2781 (4)	0.6636 (3)	0.78360 (19)	0.0171 (5)	
H7	0.211 (4)	0.629 (3)	0.8726 (19)	0.0205 (5)*	
C8	0.1121 (4)	0.7774 (3)	0.72290 (19)	0.0178 (5)	
H8	−0.086 (4)	0.841 (3)	0.7634 (19)	0.0214 (5)*	
C9	0.1997 (4)	0.8101 (3)	0.61046 (18)	0.0152 (4)	
H9	0.075 (4)	0.900 (3)	0.5609 (19)	0.0182 (5)*	

### 3-(Bromomethyl)-6-phenyl-1,2,4,5-tetrazine (3) Atomic displacement parameters (Å^2^)

**Table:**

	*U*^11^	*U*^22^	*U*^33^	*U*^12^	*U*^13^	*U*^23^
Br1	0.02876 (14)	0.02440 (14)	0.01709 (13)	−0.00081 (10)	−0.00401 (9)	−0.00149 (9)
N1	0.0139 (9)	0.0189 (10)	0.0180 (10)	0.0008 (7)	−0.0008 (7)	−0.0004 (8)
N2	0.0124 (9)	0.0200 (10)	0.0189 (10)	0.0018 (8)	−0.0022 (7)	0.0004 (8)
N3	0.0134 (9)	0.0171 (10)	0.0194 (10)	0.0015 (7)	−0.0024 (7)	0.0004 (8)
N4	0.0159 (10)	0.0192 (10)	0.0205 (10)	0.0012 (8)	−0.0030 (7)	0.0022 (8)
C1	0.0201 (12)	0.0232 (13)	0.0195 (12)	−0.0036 (10)	−0.0020 (9)	0.0040 (10)
C2	0.0149 (11)	0.0156 (11)	0.0171 (11)	−0.0015 (8)	−0.0036 (8)	0.0004 (9)
C3	0.0116 (10)	0.0135 (11)	0.0180 (11)	−0.0010 (8)	−0.0032 (8)	−0.0010 (9)
C4	0.0121 (10)	0.0098 (10)	0.0183 (11)	−0.0003 (8)	−0.0041 (8)	−0.0015 (8)
C5	0.0114 (10)	0.0134 (11)	0.0181 (11)	0.0019 (8)	−0.0023 (8)	−0.0008 (9)
C6	0.0145 (11)	0.0153 (11)	0.0214 (12)	0.0012 (9)	−0.0045 (9)	−0.0006 (9)
C7	0.0166 (11)	0.0153 (11)	0.0186 (12)	−0.0013 (9)	−0.0023 (9)	0.0012 (9)
C8	0.0129 (11)	0.0181 (12)	0.0206 (12)	0.0003 (9)	−0.0008 (9)	−0.0009 (9)
C9	0.0115 (10)	0.0152 (11)	0.0179 (11)	−0.0000 (8)	−0.0021 (8)	0.0004 (9)

### 3-(Bromomethyl)-6-phenyl-1,2,4,5-tetrazine (3) Geometric parameters (Å, º)

**Table:**

Br1—C1	1.962 (2)	C4—C5	1.404 (3)
N1—N2	1.318 (2)	C4—C9	1.399 (3)
N1—C2	1.338 (3)	C5—H5	1.09 (2)
N2—C3	1.348 (3)	C5—C6	1.397 (3)
N3—N4	1.314 (2)	C6—H6	1.08 (2)
N3—C3	1.351 (3)	C6—C7	1.397 (3)
N4—C2	1.345 (3)	C7—H7	1.12 (2)
C1—H1a	1.13 (2)	C7—C8	1.397 (3)
C1—H1b	1.15 (3)	C8—H8	1.09 (2)
C1—C2	1.489 (3)	C8—C9	1.394 (3)
C3—C4	1.478 (3)	C9—H9	1.10 (2)
			
C2—N1—N2	117.68 (17)	C9—C4—C3	120.19 (18)
C3—N2—N1	117.92 (17)	C9—C4—C5	119.94 (19)
C3—N3—N4	118.14 (18)	H5—C5—C4	122.4 (11)
C2—N4—N3	117.46 (18)	C6—C5—C4	119.76 (19)
H1a—C1—Br1	109.3 (12)	C6—C5—H5	117.8 (11)
H1b—C1—Br1	105.8 (12)	H6—C6—C5	118.5 (12)
H1b—C1—H1a	115.0 (17)	C7—C6—C5	119.9 (2)
C2—C1—Br1	110.83 (15)	C7—C6—H6	121.6 (12)
C2—C1—H1a	107.5 (12)	H7—C7—C6	118.0 (12)
C2—C1—H1b	108.4 (12)	C8—C7—C6	120.4 (2)
N4—C2—N1	125.0 (2)	C8—C7—H7	121.5 (12)
C1—C2—N1	117.44 (19)	H8—C8—C7	120.2 (12)
C1—C2—N4	117.57 (19)	C9—C8—C7	119.8 (2)
N3—C3—N2	123.82 (19)	C9—C8—H8	120.0 (12)
C4—C3—N2	118.23 (18)	C8—C9—C4	120.18 (19)
C4—C3—N3	117.95 (18)	H9—C9—C4	118.2 (12)
C5—C4—C3	119.87 (18)	H9—C9—C8	121.6 (12)
			
Br1—C1—C2—N1	107.43 (16)	N3—C3—C4—C9	−2.1 (2)
Br1—C1—C2—N4	−74.63 (17)	N4—N3—C3—C4	−179.36 (18)
N1—N2—C3—N3	−0.9 (2)	C2—N1—N2—C3	−0.2 (2)
N1—N2—C3—C4	179.59 (18)	C2—N4—N3—C3	−0.3 (2)
N1—C2—N4—N3	−0.8 (2)	C3—C4—C5—C6	179.94 (19)
N2—N1—C2—N4	1.0 (2)	C3—C4—C9—C8	−179.63 (19)
N2—N1—C2—C1	178.79 (19)	C4—C5—C6—C7	0.3 (2)
N2—C3—N3—N4	1.1 (2)	C4—C9—C8—C7	−0.9 (2)
N2—C3—C4—C5	−2.9 (2)	C5—C4—C9—C8	0.7 (2)
N2—C3—C4—C9	177.44 (19)	C5—C6—C7—C8	−0.5 (2)
N3—N4—C2—C1	−178.56 (19)	C6—C5—C4—C9	−0.4 (2)
N3—C3—C4—C5	177.48 (18)	C6—C7—C8—C9	0.8 (2)

## References

[bb1] Baalmann, M., Ziegler, M. J., Werther, P., Wilhelm, J. & Wombacher, R. (2019). *Bioconjugate Chem.* **30**, 1405–1414. 10.1021/acs.bioconjchem.9b00157 30883100

[bb2] Bourhis, L. J., Dolomanov, O. V., Gildea, R. J., Howard, J. A. K. & Puschmann, H. (2015). *Acta Cryst.* A**71**, 59–75. 10.1107/S2053273314022207 PMC4283469 25537389

[bb3] Dolomanov, O. V., Bourhis, L. J., Gildea, R. J., Howard, J. A. K. & Puschmann, H. (2009). *J. Appl. Cryst.* **42**, 339–341.

[bb4] Fulmer, G. R., Miller, A. J. M., Sherden, N. H., Gottlieb, H. E., Nudelman, A., Stoltz, B. M., Bercaw, J. E. & Goldberg, K. I. (2010). *Organometallics* **29**, 2176–2179.

[bb5] Groom, C. R., Bruno, I. J., Lightfoot, M. P. & Ward, S. C. (2016). *Acta Cryst.* B**72**, 171–179. 10.1107/S2052520616003954 PMC4822653 27048719

[bb6] Hu, W.-X. & Xu, F. (2008). *J. Heterocycl. Chem.* **45**, 1745–1750.

[bb7] Huber, R. G., Margreiter, M. A., Fuchs, J. E., von Grafenstein, S., Tautermann, C. S., Liedl, K. R. & Fox, T. (2014). *J. Chem. Inf. Model.* **54**, 1371–1379. 10.1021/ci500183u PMC4037317 24773380

[bb8] Kleemiss, F., Dolomanov, O. V., Bodensteiner, M., Peyerimhoff, N., Midgley, L., Bourhis, L. J., Genoni, A., Malaspina, L. A., Jayatilaka, D., Spencer, J. L., White, F., Grundkötter-Stock, B., Steinhauer, S., Lentz, D., Puschmann, H. & Grabowsky, S. (2021). *Chem. Sci.* **12**, 1675–1692. 10.1039/d0sc05526c PMC8179328 34163928

[bb9] Lang, S. A., Johnson, B. D. & Cohen, E. (1975). *J. Heterocycl. Chem.* **12**, 1143–1153.

[bb10] Neese, F. (2022). *WIREs Comput. Mol. Sci.* **12**, e1606.

[bb11] Pinner, A. (1893). *Ber. Dtsch. Chem. Ges.* **26**, 2126–2135.

[bb12] Rigaku OD (2025). *CrysAlis PRO*. Rigaku Oxford Diffraction, Yarnton, England.

[bb13] Sheldrick, G. M. (2015). *Acta Cryst.* A**71**, 3–8.

[bb14] Tu, J., Svatunek, D., Parvez, S., Eckvahl, H. J., Xu, M., Peterson, R. T., Houk, K. N. & Franzini, R. M. (2020). *Chem. Sci.* **11**, 169–179. 10.1039/c9sc04649f PMC7012038 32110368

[bb15] Voss, J., Stammler, H. G. & Sewald, N. (2025). *Acta Cryst.* E**81**, 164–168. 10.1107/S2056989025000441 PMC11799789 39927388

[bb16] Wang, Y., Shen, G., Li, J., Mao, W., Sun, H., Feng, P. & Wu, H. (2022). *Org. Lett.* **24**, 5293–5297. 10.1021/acs.orglett.2c01873 35848542

[bb17] Westrip, S. P. (2010). *J. Appl. Cryst.* **43**, 920–925.

[bb18] Xu, F. & Hu, W.-X. (2007). *Acta Cryst.* E**63**, o2397.

[bb19] Yim, J., Park, J., Lee, Y. & Park, J. (2026). *Chem. Eur. J.* **32**, e71028. 10.1002/chem.71028 PMC13356370 42028900

[bb20] Zhao, G., Li, Z., Zhang, R., Zhou, L., Zhao, H. & Jiang, H. (2022). *Front. Mol. Biosci.* **9**, 1055823. 10.3389/fmolb.2022.1055823 PMC9709424 36465558

